# Are rural residents missing out on teaching? A comparison of teaching opportunities for urban and rural family medicine residents at the University of Manitoba

**Published:** 2018-03-27

**Authors:** Aaron Jattan, Charles Penner, Joanne Maier, Bruce Martin

**Affiliations:** 1 University of Manitoba, Manitoba, Canada

## Abstract

**Background:**

The scholar competency of the CanMEDS-FM framework requires residents to develop teaching skills, and with the number of rural residency positions tripling over the last decade, it is essential for residency programs to provide rural residents with teaching opportunities. The purpose of this study was to investigate the differences in teaching opportunities offered to urban and rural family medicine residents at the University of Manitoba.

**Methods:**

The 117 urban and rural family medicine residents were surveyed to quantify their interactions with medical students. Specific respondents were interviewed to provide context to the survey.

**Results:**

On family medicine, only 20% of rural residents reported frequent opportunities to informally teach, compared to 57% of urban residents. Similarly, 86% of urban residents reported organized teaching opportunities compared to only 5% of rural residents. Residents placed high value in receiving additional teaching opportunities.

**Conclusion:**

This study suggests that there are fewer teaching opportunities for rural family medicine residents compared to urban residents at the University of Manitoba. Given the small sample size, a larger study could determine whether this trend exists across the country. It will be incumbent on residency programs to ensure rural residents have the opportunities to become competent educators.

## Introduction

Distributed medical education in rural communities continues to grow in an effort to meet the demands of the Canadian population. The Society of Rural Physicians of Canada reported that the number of rural focused family medicine residency spots increased to 446 in 2013 from 144 spots in 2009.^1^ Wenghofer et al. showed in a recent study that residents who pursue and train in rural centers are more likely to practice in similar rural centers.^2^ Furthermore, educating and training medical students and residents in rural centers has been shown to be a successful method of recruitment.^3^ This is a positive finding in our collective efforts to better distribute physicians to rural communities in need of physicians.

One of the challenges facing medical schools throughout the growth into these rural communities has been preceptor recruitment. A study by Piggot et al. exploring barriers facing physicians in rural communities who were not actively precepting revealed that lack of teaching experience was a significant factor.^4^ One physician stated that, “I have never been formally taught how to teach…I only have training as a doctor. I am not an educator.” However, if we are to meet the challenges of physician recruitment in these communities, it is critical that we train skilled educators who can inspire and teach future generations of rural physicians.

In 2009, the Canadian College of Family Physicians released the CanMEDS-Family Medicine framework to guide the design and delivery of residency programs across the country.^5^ The framework consists of the following roles: family medicine expert, communicator, collaborator, manager, health advocate, scholar, and professional. The *scholar* component of this framework mandates that residents “facilitate the education of patients, families [and] trainees…” Additionally, the guideline directs that residents develop effective teaching strategies and provide effective feedback to learners. Furthermore, the CFPC Red Book which outlines accreditation standards for all family medicine programs across the country unequivocally states that, “residents must be given opportunities to develop effective teaching skills through organized activities focused on teaching techniques [and] should have opportunities to teach and to become role models to junior residents and medical students.”^6^ Several studies have also shown that residents perceive teaching students as a significant responsibility throughout their residency.^7,8^

There is a paucity of data exploring the quantity and quality of clinical teaching training that rural family medicine residents receive compared to their urban colleagues. It is with this in mind that we undertook a study to evaluate the differences in clinical teaching opportunities between urban and rural family medicine residents at the University of Manitoba. The University of Manitoba family medicine residency competency framework highlights the requirements outlined by the CFPC Red Book stating that all residents will develop effective learner-centred teaching skills and will be able provide effective feedback to learners by the conclusion of their residency.^9^ The predominant interaction we explored was those that residents had with medical students. It was our hope that if a disparity were identified, that greater emphasis could be placed on ensuring that rural family medicine residents receive appropriate training and opportunities to develop their teaching skills.

## Methods

### Literature search

We conducted an online search in University of Manitoba OneStop Search, PubMed, and Scopus. The search terms included medical resident (or post-graduate medical education), teaching, rural and urban. Specific MeSH terms of [Education, Medical] and [Education, Medical, Graduate] were also used. Prominent medical education journals were also searched separately, namely the Canadian Medical Education Journal and Medical Teacher.

### Study content

This study was a mixed method study consisting of a survey and optional interview. The study population included the 117 family medicine residents enrolled at the University of Manitoba during the academic year of 2015-2016. The sample population included both PGY1 and PGY2 residents. No exclusion criteria were applied.

The survey was constructed with the assistance of two deans within the Faculty of Medicine at the University of Manitoba. The aim of the survey was to quantitatively and qualitatively determine the teaching opportunities that exist for family medicine residents while on-service and off-service. Demographic data included the level of the resident and the program they belonged to (urban vs. rural).

The quantitative component of the questionnaire utilized a five-point Likert scale to identify the frequency of interactions between residents and medical students. The Likert scale options included daily interactions, weekly interactions, monthly interactions, yearly interactions, and no interactions. The questions were separated based on whether the interaction was off-service or on-service. Further distinctions were based on whether the interaction was formal or informal. It was indicated to the residents that formal teaching involved activities that were scheduled by the faculty while informal teaching involved incidental teaching such as bedside teaching on the ward.

The survey then asked residents to specifically identify the type of interaction that they had with medical students. Specific examples were chosen that residents could select from and a fillable box was included such that residents could identify any activity not listed.

The conclusion of the survey asked residents to quantify the value they placed in teaching medical students and their perceptions of the online teaching courses offered at the University of Manitoba.

Additionally, the survey offered the opportunity for respondents to leave their email address if they wished to participate in a brief follow-up interview. We then arranged interviews with those who elected to participate. The aim of the interviews was to identify different themes between the urban and rural residents concerning their opportunities to develop clinical teaching skills.

### Delivery

We distributed he survey via email to all family medicine residents in May 2016. The email included details of the project, a disclaimer that the survey was voluntary and an opportunity to decline if they so wished. Using the link provided, participants could access the survey online, which was hosted securely on FluidSurveys. A reminder email was sent an additional two times at monthly intervals.

As noted, the last question of the survey asked participants to leave their email address should they be willing to participate in a brief interview to further elaborate on their survey answers. All eight individuals who wished to participate in the survey were emailed in order to set-up face-to-face or phone interviews. The primary investigator conducted the interviews and used the same interview questionnaire in each case. The questions were designed to allow participants to elaborate on the survey responses by providing specific examples and to give examples to barriers that they may have faced with respect to teaching. The interviews were recorded without any identifying data and were stored on a secure hard-drive. The only identifying data recorded was whether the interviewee was an urban or rural family medicine resident.

### Analysis

Given the low response rate, the results of the quantitative survey were categorized into buckets indicating *frequent* teaching opportunities (daily, weekly), *infrequent* opportunities (monthly, yearly) or *no teaching opportunities*. The results were then analyzed.

The interviews themselves were transcribed and common themes were extracted by the primary investigator.

### Approvals

The study design was approved by the University of Manitoba Health Research Ethics Board.

## Results

### Study participants

The survey was distributed to the 117 family medicine residents who were registered in the Family Medicine program at the University of Manitoba in May 2016. The survey was answered by 27 residents for a total response rate of 23.1%. Survey study participants are characterized in Table 1.

**Table 1:** Characteristics of study participants

Location			
	Urban	Rural	Total
Level			
PGY1	3	14	17
PGY2	4	6	10
Total	7	20	27

### Teaching opportunities on family medicine time

Given the low response rate, we grouped survey responses with respect to quantity of teaching opportunities into three buckets: frequent, infrequent, and no teaching opportunities.

Almost all (93%) of the residents stated that there were medical students present during their family medicine rotation at their home site.

**Figure 1:**
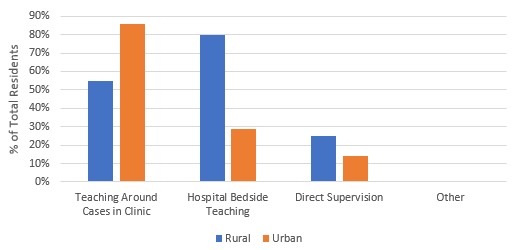
Informal teaching opportunities on family medicine

Fifty-seven percent (n=4) of urban residents felt they had frequent opportunities to teach informally compared to just 20% (n=4) of rural residents while on family medicine block time. The types of informal teaching opportunities are illustrated in Figure 1.

**Figure 2:**
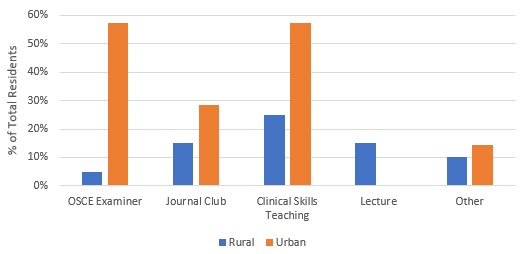
Formal teaching opportunities on family medicine

With respect to formal teaching opportunities during family medicine block time, 50% of rural family medicine residents (n=10) stated no opportunities to teach compared to just 14% (n=1) of urban residents. The types of formal teaching that were available to residents are illustrated in Figure 2.

### Teaching opportunities while off-service

**Figure 3:**
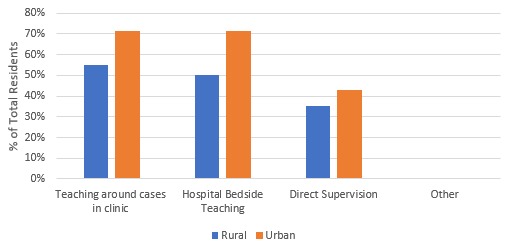
Informal teaching opportunities while off-service

All urban family medicine residents were exposed to medical students on off-service rotations compared to only 75% (n=15) of rural family medicine residents. While off-service, all urban residents (n=7) had the opportunity to teach informally (43% frequently, 57% infrequently) while 70% of rural residents (n=14) also had the opportunity to teach (45% frequently, 25% infrequently). Figure 3 outlines the types of informal teaching opportunities available to residents.

**Figure 4:**
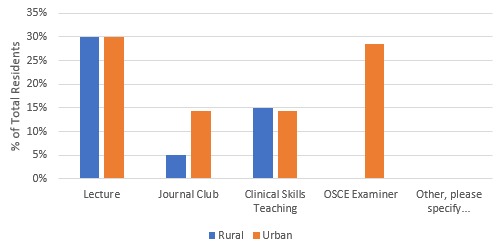
Formal teaching opportunities while off-service

Concerning formal teaching opportunities, the majority of residents (57% urban [n=4], 55% rural [n=11]) had no opportunities to teach. Figure 4 outlines details of the types of teaching opportunity.

### Value of online modules

The University of Manitoba provides two online modules as a part of the Teaching Development Program (TDP) in order to provide residents with teaching tools and skills. These modules consist of both content quizzes and self-reflection exercises. Completion is mandatory by the conclusion of a family medicine residency at the University of Manitoba.

The majority of residents who had completed these modules while the survey was open (55%, n=11) felt that these modules had some relevance while 25% (n=5) felt that these had minimal to no relevance when it came to their perceived role as teachers in their family medicine residency.

Seventy-percent (n=18) of all residents surveyed placed high value in increasing the amount of teaching opportunities available to them during family medicine residents.

### Interview themes

We interviewed five rural residents and three urban residents.

While on family medicine block time, rural residents commonly cited no opportunities to formally teach. Furthermore, they cited while medical students were present at their clinic, there were limited opportunities to interact informally with them in a teaching capacity. Urban residents interviewed cited both formal and informal opportunities to teach. Urban residents commonly cited clinical teaching opportunities which were scheduled for them as well as opportunities to informally teaching during on-call shifts.

While off-service, both urban and rural residents commonly cited no formal or scheduled opportunities to teach medical students. However, both urban and rural residents spoke of opportunities on obstetrics and internal medicine to supervise directly medical students, specifically while on call.

All residents interviewed placed a high value on teaching medical students and desired more teaching opportunities. However, it was commonly expressed that there was a need for more feedback on clinical teaching skills.

## Discussion

This study identified that there is a probable disparity in teaching opportunities for the rural family medicine residents compared to the urban family medicine residents at the University of Manitoba though with such a small sample size the differences did not reach statistical significance.

This outcome was primarily seen while family medicine residents were on-service and included both informal and formal teaching. In general, the disparity in formal opportunities could be explained by the fact that medical students complete the bulk of their training in urban centers where they also attend resident-led activities such as clinical-teaching sessions and small-group learning. The interviews supported these results as urban residents cited opportunities to have scheduled clinical skills teaching sessions. A rural resident who was interviewed stated that they were offered the opportunity to participate in clinical skills teaching with medical students but they would have had to commute three hours to an urban center in order to do so.

A contributing factor to the lack of informal teaching opportunities for rural residents is that most learners work one-on-one with preceptors in rural centers. There are similarly fewer clinical teaching unit teams in rural centers where a resident would have direct supervision over a medical student. It makes sense that there would be a lack of emphasis on resident teaching in rural centers. One rural resident cited an example while on family medicine of being “scolded” when attempting to teach a medical student. The resident attempted to have the medical student see an interesting patient on the ward but was discouraged by her preceptor as the medical student was assigned to a different preceptor. In this specific rural setting, like many, it is uncommon for more than one learner to be assigned to a preceptor the resident stated.

However, it did appear that urban and rural residents had similar teaching opportunities while off-service. Forty-five percent of rural residents and 43% of urban residents cited frequent opportunities to teach informally while off-service. This was corroborated by the interviews in which both urban and rural residents cited opportunities to directly supervise or conduct bedside teaching with medical students, specifically while rotating through internal medicine and obstetrics. This finding may be because many rural residents attend their off-service rotations in urban centers with other urban residents.

Unfortunately, the study in general is limited by the small sample size and the number of respondents, which made it difficult to perform a more robust statistical analysis. Additionally, the convenience sample obtained was likely a segment of the population that was interested in the resident’s role as a teacher. For example, those who cited frequent informal opportunities to teach were likely motivated to seek out such opportunities. In addition, the study included PGY1s at the end of their first year who potentially would have had teaching opportunities in their second year.

The University of Manitoba, like other schools, offer online clinical teaching modules in order to fulfill accreditation guidelines that all residency programs provide residents with clinical teaching skills. Lacasse et al. studied the preferred curriculum format for learning clinical teaching skills and found that interactive online learning modules were one of the least preferred formats for residents.^10^ The survey here showed that residents did not place a high value on the online teaching modules offered and 70% said that they would place high value on increased teaching opportunities. Most interview participants felt that observed feedback of their clinical teaching skills would be of particular value.

Ideally, both urban and rural residents would have opportunities to participate in scheduled, observed teaching with feedback sessions. This could be done in addition to online modules that could be used to teach core concepts. Furthermore, it would be incumbent on rural programs to ensure that residents and medical students be given the opportunity to interact such that rural residents can develop their teaching skills while working in a family medicine setting. This will undoubtedly be a challenge given the inherent differences in the structures of rural and urban training programs but will be critical in supporting the pipeline of rural family medicine educators.

### Conclusion

Distributed medical education continues to expand throughout Canada in an effort to meet the demands of the population and to provide medical care to the underserviced population that reside away from large urban centers. In order to strengthen the pipeline to these rural communities, it is essential that we train physicians in these communities such that they are comfortable teaching. What this study showed is that there are likely fewer teaching opportunities for rural residents to teach and interact with medical students compared to their urban counterparts. There are clear reasons for this given that there are often fewer learners in rural environments and that learners typically work one-on-one with preceptors. However, it will be essential for distributed residency programs to study how best to enhance teaching opportunities for residents given these limitations. Future research should attempt to elucidate whether this trend exists across the country and what method of learning to teach would be an improvement over on-line modules.
